# TENASCIN-Dependent Mesenchymal–Epithelial Interactions Associated with Interstitial Microenvironment Remodeling in Cattle-Yak Lung: Insights from Single-Nucleus Transcriptomics

**DOI:** 10.3390/ijms27198531

**Published:** 2026-09-24

**Authors:** Qianyun Ge, Jie Pei, Lin Xiong, Xiaoyun Wu, Xian Guo

**Affiliations:** 1 Key Laboratory of Yak Breeding of Gansu Province, Lanzhou Institute of Husbandry and Pharmaceutical Sciences, Chinese Academy of Agricultural Sciences, Lanzhou 730050, China; geqianyun@caas.cn (Q.G.); peijie@caas.cn (J.P.); xionglin@caas.cn (L.X.); wuxiaoyun@caas.cn (X.W.); 2 Key Laboratory of Animal Genetics and Breeding on Tibetan Plateau, Ministry of Agriculture and Rural Affairs, Lanzhou 730050, China

**Keywords:** cattle-yak, snRNA-seq, lung interstitial remodeling, TENASCIN signaling pathway, hypoxia adaptation

## Abstract

Cattle-yak, a hybrid of cattle and yak, exhibits improved milk yield, accelerated growth, and strong physiological adaptation to high-altitude environments, conferring substantial commercial value. However, the transcriptomic basis of hypoxia adaptation in cattle-yak remains poorly characterized at the single-nucleus level. More than 20,000 cells were profiled, and subpopulations representing the major lung cell types in yak and cattle-yak were identified. A higher proportion of mesenchymal cells were observed in cattle-yak compared with yak. Tnc ligands expressed by alveolar fibroblasts, alveolar lipofibroblasts, and ductal myofibroblasts interact with the epithelial Itgav + Itgb6 and Itgav + Itgb3 receptors through the TENASCIN signaling pathway, thereby potentially contributing to lung morphogenesis in cattle-yak. These findings suggest that cattle-yaks have an enhanced capacity for lung interstitial remodeling, which may underlie their hybrid advantage in adapting to high-altitude hypoxia while maintaining growth and production performance. In addition, genes associated with lung mesenchymal cell remodeling and differences in lung tissue were identified. This study first delineated the molecular characteristics of lung cell types in cattle-yak and addressed the gap in comparative single-nucleus analyses between yak and cattle-yak lung tissues. Collectively, these results advance the understanding of high-altitude adaptation in highland cattle breeds and provide valuable insights into animal husbandry in such environments.

## 1. Introduction

The Tibetan Plateau is characterized by harsh environmental conditions, including low temperatures, reduced partial pressure of oxygen (PO2), and intense ultraviolet (UV) radiation, all of which pose significant challenges to both human and animal survival [1,2]. Among these factors, hypoxia remains consistent, in contrast to seasonal variations in temperature and UV exposure. Therefore, animals adapted to high-altitude environments can tolerate chronic hypoxic conditions [3,4,5].

Yaks (*Bos grunniens*), native to the Qinghai-Tibet Plateau, are important models for studying hypoxia adaptation in large mammals. Prolonged exposure to hypoxic conditions has driven the evolution of distinct anatomical and physiological traits that facilitate survival at extremely high altitudes [6,7,8]. The publication of the yak genome draft has enabled extensive genomic investigations into high-altitude adaptation [9]. These studies have identified adaptive signatures associated with oxygen transport and hypoxia resistance, energy metabolism, deoxyribonucleic acid (DNA) repair, and cellular survival and proliferation pathways [9,10]. In contrast, closely related and cross-fertile taurine cattle (*B. taurus*) exhibit severe pulmonary hypertension when raised in yak habitats [11]. Within the Bos genus, cattle (*B. taurus*) and yak (*B. grunniens*) diverged evolutionarily approximately 4.9 million years ago [9]. Despite this divergence, interspecific hybridization between cattle and yak remains possible in both reciprocal crosses, although male F1 hybrids are sterile, likely due to impaired spermatogenesis [12]. Modern cattle breeds have been selected for high productivity in economically important traits, whereas yaks have undergone long-term adaptive evolution for survival in high-altitude environments [6]. However, the physiological performance and productivity of cattle decline markedly at elevations above 2500 m [13]. Consequently, cattle-yak hybrids exhibit significantly improved milk yield, enhanced growth performance, and strong physiological adaptation to high-altitude environments, conferring substantial commercial value. Nevertheless, the genomic basis underlying hypoxia adaptation of cattle-yak remains poorly understood.

The lung serves as the primary interface between the organism and the external environment and, therefore, plays a central role in high-altitude adaptation, attracting considerable research interest in recent years. Plateau-adapted animals generally exhibit attenuated hypoxic pulmonary vasoconstriction, reduced pulmonary arterial remodeling, and improved metabolic efficiency compared with low-altitude species [6,14]. Comparative transcriptomic analyses across tissues in different species have shown that the lung displays pronounced adaptive transcriptional changes and a higher proportion of positively selected genes than other organs [6]. In yaks, well-developed pulmonary blood vessels enhance the oxygen exchange rate of pulmonary artery blood vessels and alleviate pulmonary arterial pressure. Despite these advances, the cellular composition and gene expression dynamics of lung tissue in high-altitude-adapted animals remain insufficiently characterized at single-nucleus resolution. Cells are the fundamental structural and functional units of all biological organisms, each of which possesses unique characteristics. Traditional bulk ribonucleic acid (RNA) sequencing and microarray approaches measure average gene expression across cell populations but cannot resolve cellular heterogeneity at the single-cell level. In contrast, single-nucleus RNA (sn-RNA) sequencing enables the profiling of gene expression in individual cells and facilitates the identification of known and novel cell types within tissues and organs at transcriptomic resolution [15,16,17]. Recently, such studies have yielded important findings in human and mouse lung systems [18,19]. A single-cell gene expression atlas of yak and taurine cattle lung tissues has been established, revealing a novel endothelial cell subtype and identifying genes and pathways associated with the development of unique structural features in the yak lung [20]. However, studies focusing on hypoxia adaptation in cattle-yaks remain limited.

A single-nucleus atlas of yak and cattle-yak lungs was constructed using snRNA-seq. Cell-type-specific marker genes and differentially expressed genes between yak and cattle-yak were identified, potentially involved in key cellular functions. Cell-cell communication networks were inferred based on highly expressed ligand–receptor pairs. Mesenchymal cell heterogeneity was further characterized based on subtype-specific gene expression. In addition, developmental trajectories of individual cell populations were inferred using pseudotime analysis. This study provides the first comprehensive molecular characterization of cattle-yak lung cell types and addresses a critical gap in single-nucleus comparative analyses between yak and cattle-yak. These findings enhance the understanding of parental contributions to the environmental adaptation of cattle-yak hybrids.

## 2. Results

### 2.1. Construction of Single-Nucleus Atlases in Yak and Cattle-Yak Lung Using snRNA-Seq

Although both yak and cattle-yak exhibit hypoxia adaptation, differences in cell population diversity and gene expression profiles at the single-nucleus level in their lungs remain largely unexplored. To address this, single-nucleus RNA sequencing was performed on lung tissue samples collected from adult yak and cattle-yak. Each sample comprised pooled lung tissue from three individuals per group. Libraries were prepared for snRNA sequencing using the 10× Chromium platform. Raw snRNA-seq reads were processed and converted into Seurat objects using the Cell Ranger workflow. Sequencing saturation of yaks and cattle-yaks was 76.7% and 74.7%, respectively, indicating that the sequencing depth reached saturation for all libraries. The number of detected genes (nFeature), unique molecular identifiers (nUMIs), and mitochondrial gene distribution ratio (percent.MT) were computed and visualized for quality control (Supplementary Figure S1). Cells with <300 or >2800 detected genes, mitochondrial gene content exceeding 20%, or UMI counts >7800 were excluded. After preprocessing and quality filtering, 21,488 cells were retained, including 10,885 domestic yak cells and 10,603 cattle-yak cells. The snRNA-seq dataset was successfully normalized and centered, and dimensionality reduction was performed for downstream analyses. The reads mapped confidently to the genome were 85.7% and 84.5%, respectively. The results indicated that the transcriptome sequencing data were of high quality and had a suitable mapping rate.

### 2.2. Cell Clustering and Marker Gene Selection

After quality filtering and integration of both datasets, dimensionality reduction was performed using Uniform Manifold Approximation and Projection (UMAP) to group cells with similar gene expression. Following a coarse clustering of the transcriptomic data at a resolution of 0.5, the dataset was partitioned into 24 and 25 cell clusters in yak (0–23) and cattle-yak (0–24), respectively (Figure 1A). The tSNE was provided in Figure S2. Notably, cluster 24 was exclusively present in cattle-yak cells. DEGs for each cluster were identified using Wilcoxon rank-sum testing, and the DEGs per cluster were selected based on adjusted *p*-values (Supplementary Table S1). A heat map shows the expression patterns of the top five most variable genes per cluster (Figure 1B). A dot plot illustrates the expression levels and percentages of representative genes across clusters (Figure 1C).

### 2.3. Cell Type Identification

We used the known cell type markers from published literature for cell annotation; the specific work involved examining the high expression of known markers in each cluster in the single-nucleus data to determine the cell type to which the cluster belonged. Five major cell types were identified in the yak lung, including epithelial cells (25.2%, four clusters), endothelial cells (14.9%, four clusters), mesenchymal cells (8.1%, three clusters), immune cells (51.6%, twelve clusters), and unknown cells (0.2%, one cluster) (Figure 2A). Similarly, the same five cell types were identified in cattle-yak lung, including epithelial cells (25.3%, four clusters), endothelial cells (16.1%, four clusters), mesenchymal cells (12.7%, three clusters), immune cells (45.7%, thirteen clusters), and unknown cells (0.2%, one cluster) (Figure 2A). Detailed annotations are provided in Supplementary Table S2. These four cell types were also observed in mouse lungs, indicating that the principal cellular components of lung tissue were preserved across species [21]. The distribution of cell numbers across clusters and cell types is illustrated in a balloon plot (Figure 2B). Differences in the cellular frequency of five cell types between yak and cattle-yak are shown in Figure 2C. The DEGs for each cell type were selected based on their adjusted *p*-values (Supplementary Table S3). Notably, mesenchymal cells had the greatest differences in cellular frequency and the most DEGs. Expression scores and percentages of signature genes across cell types are visualized using a dot plot (Figure 2D), indicating cell type-specific gene expression signatures. The expression patterns of the top five DEGs per cell type are shown in a heat map (Figure 2E).

### 2.4. Functional Enrichment Analysis of the Lung

GO and KEGG enrichment analyses were performed using DEGs identified within each cell type (Supplementary Table S4). The GO enrichment results are summarized in bubble plots based on the top five biological processes with the highest false discovery rate (FDR) enrichment for each cell type, highlighting their principal biological functional roles (Figure 3A). Endothelial cell DEGs are highly expressed in the regulation of cell migration and blood vessel development. DEGs were associated with actin filament-based processes, plasma membrane-bounded cell projection organization, and cellular or subcellular movement in epithelial cells. Immune cells are enriched in the immune system. Mesenchymal cell DEGs were associated with anatomical structure morphogenesis and cellular movement. The anatomical structure development and cytoskeleton organization of the unknown cells were enriched. These findings are consistent with the functional identities of each cell type.

Beyond variations in cellular composition, differences in gene expression within the same cell population may also underlie the divergence between yak and cattle-yak. The mesenchymal cells exhibited 1590 DEGs (Figure S3, Supplementary Table S5). Functional gene enrichment of these DEGs highlighted pathways associated with cellular protein metabolism. Epithelial, immune, and endothelial cells were predominantly enriched in “intracellular signal transduction,” “cellular responses to chemical stimuli,” and “cell migration and motility,” respectively. (Figure 3B, Supplementary Table S6). Core genes (*ACTB*, *CTNNB1*, and *UBB*) identified from the top five enriched biological process (BP) terms for DEGs between yak and cattle-yak were analyzed using STRING (12.0) and visualized using Cytoscape (3.10.4) (Figure 3C). STRING analysis parameters: Network Type: full STRING network; Required score: medium confidence (0.400); FDR stringency: medium (5 percent).

### 2.5. Ligand–Receptor Analysis Reveals Dysregulated TENASCIN Signaling in Cattle-Yak Lung

CellChat (v2.1.2) was used to establish intercellular communication networks to delineate interactions among the five lung cell types [22]. The results of CellChat significant receptor pair cell communication are provided in Table S7. Mesenchymal cells exhibited prominent interaction with epithelial cells (Figure 4A,B). To characterize communication patterns driven by differentially expressed ligand–receptor pairs between mesenchymal and epithelial cells, statistical inference was conducted using CellChat with an emphasis on predicted cell–cell contact pathways specifically enriched in cattle-yak cells. Twenty-two signaling pathways exhibited the highest statistical measurements of information flow in cattle-yaks compared with yaks (Figure 4C). To assess how these inferred interactions could influence epithelial cell behavior, ligand predictions originating from mesenchymal cells and receptor predictions from epithelial cells were specifically examined. Among the qualifying pathways, TENASCIN signaling emerged as a key candidate (Figure 4D). Notably, within the established ligand–receptor pairs, Tnc ligands engaged the multimeric receptors Itgav + Itgb6 and Itgav + Itgb3 to mediate TENASCIN signaling (Figure 4E, Supplementary Table S8).

### 2.6. Potential Functional Heterogeneity of Mesenchymal Cells

Considering the greatest proportion change, massive differential-gene signature, and strong intercellular communication function, we prioritized mesenchymal cells for subsequent downstream analyses. Mesenchymal cells were subdivided into seven different subtypes following clustering (Figure 5A). DEGs for each subtype were successfully identified (Supplementary Table S9). The expression patterns of the top five highly variable DEGs per subtype are presented in a heat map (Figure 5B) and a dot plot (Figure 5C). GO enrichment analysis of subtype-specific DEGs was performed (Supplementary Table S10) and summarized in a bubble plot (Figure 5D). Subtype 0 DEGs were enriched in cell migration regulation. Subtype 2 showed enrichment in cell migration regulation and anatomical structure morphogenesis. Genes highly expressed in subtype 1 were predominantly associated with multicellular organism development. Subtypes 5 and 6 were mainly enriched in tissue and blood vessel development, respectively. Subtype 4 was associated with translation and peptide metabolic processes, whereas subtype 3 was associated with immune system processes.

### 2.7. Pseudotime Expression Patterns of Representative Genes in Mesenchymal Cells

Pseudotime analysis was performed using Monocle 2, focusing on mesenchymal cells. Analyses performed separately for yak or cattle-yak mesenchymal cells showed similar developmental trajectories; therefore, all 2234 mesenchymal cells were combined for joint analysis (Figure 6A).

A total of 1234 dynamically expressed genes were identified and classified into five expression patterns (Supplementary Table S11). Genes with late-stage expression are potential regulators of mesenchymal cell differentiation. The sustained expression of these genes may contribute to cellular state transitions during mesenchymal evolution. Genes in cluster 2 showed continuously increasing expression along pseudotime and were enriched in GO terms related to localization regulation and stimulus response, and KEGG pathways associated with vascular smooth muscle contraction. *ACTB* was also identified as a core regulatory gene potentially involved in mesenchymal cell differentiation by analyzing the top 5 GO terms.

## 3. Discussion

Single-nucleus RNA-seq analyses have enabled the comprehensive characterization of cellular components and developmental trajectories in human, mouse, and other animal lung tissue [21,23]. A single-cell atlas of lung tissues from yak and taurine cattle identified a yak-specific endothelial cell subtype. It revealed pathways associated with medial thickness and elastic fiber formation in yak lungs. However, single-nucleus-level data for cattle-yak lung and comparative analysis between yak and cattle-yak remain lacking. In this study, for the first time, lung cell landscapes of yak and cattle-yak were mapped using single-nucleus technology.

To investigate differences in cellular composition and gene expression features, integrated analysis of snRNA-seq from yak and cattle-yak was performed. The UMAP analysis identified five cell types at a resolution of 0.5 (Figure 2A). Five principal cell types, including epithelial cells (25.2%, four clusters), endothelial cells (14.9%, four clusters), mesenchymal cells (8.1%, three clusters), immune cells (51.6%, twelve clusters), and unknown cells (0.2%, one cluster), were detected in the yak lung (Figure 2A; Supplementary Table S2). A similar cellular composition was observed in cattle-yak lung, comprising epithelial cells (25.3%, four clusters), endothelial cells (16.1%, four clusters), mesenchymal cells (12.7%, three clusters), immune cells (45.7%, thirteen clusters), and unknown cells (0.2%, one cluster) (Figure 2A; Supplementary Table S2). Notably, the proportions of mesenchymal and immune cells in the lungs have the greatest difference between yak and cattle-yak (Figure 2C). The observed difference in immune cell numbers may be attributed to the long-term adaptation of yaks to high-altitude environments, which may involve the activation of numerous inflammatory response pathways and subsequent enhancement of innate and acquired immune responses [24,25,26]. Cluster 24 of cattle-yaks was classified as goblet cells based on the marker genes *BPIFB1*, *LTF,* and *SPDEF,* and may be involved in the cellular response to chemical stimuli in cattle-yaks. In addition to differences in cellular composition, differential gene expression within the same cell population may also contribute to the divergence between yak and cattle-yak populations. Mesenchymal cells not only show the greatest differences in cellular frequency but also have the most DEGs (1590 DEGs). Functional enrichment analysis of these DEGs identified pathways associated with cellular protein metabolic processes and anatomical structure morphogenesis (Figure 3B).

These findings indicate that cattle-yaks have enhanced metabolic activity and cellular activation associated with interstitial lung microenvironment remodeling. Enrichment of cellular protein metabolic processes indicates increased activity in protein synthesis, folding, degradation, and energy metabolism, providing the molecular basis for the massive synthesis of extracellular matrix components, cytoskeletal proteins, and stress-related proteins required to maintain structural and functional stability of the lung interstitium under hypoxic conditions [27]. Concurrently, the enrichment of anatomical structure morphogenesis indicates the active involvement of mesenchymal cells in lung tissue organization, the orderly deposition of extracellular matrix, and remodeling of tissue morphology [28]. Through regulation of cell migration and extracellular matrix assembly, these processes may contribute to optimized alveolar septal architecture, enhanced lung tissue elasticity, and improved integrity of the gas–blood barrier, thereby contributing to hypoxia adaptation. Collectively, these coordinated pathways represent a key molecular basis for cattle-yak high-altitude adaptation. Compared with the “lightweight” lung structure of yak [29], which is optimized for efficient oxygen diffusion, cattle-yak exhibited enhanced lung interstitial remodeling capacity and a more developed stromal support network. This may reflect a hybrid advantage that supports adaptation to hypoxic environments while simultaneously maintaining growth and production performance, providing important insights into the physiological mechanisms underlying high-altitude adaptation in cattle-yak.

Further analysis demonstrates that *ACTB*, *CTNNB1*, and *UBB* may function as hub regulatory genes in mesenchymal cell remodeling in cattle-yak lung. *ACTB* encodes β-actin, a highly conserved cytoskeletal protein widely expressed in eukaryotic cells. It is involved in maintaining cellular and tissue architecture, regulating cell migration, division, growth, signal transmission, and cytoskeletal organization [30]. *ACTB* gene knockout in mice results in unexplained embryonic defects, underscoring its essential role in fundamental cellular functions [31]. *CTNNB1* encodes β-catenin, a key component of the Wnt signaling pathway, which mediates cell adhesion and transcriptional regulation and plays critical roles in cell proliferation, migration, and tumor progression [32]. *UBB* encodes ubiquitin B, a critical regulator of ubiquitin-mediated protein degradation and proteostasis, ensuring the removal of misfolded proteins and maintaining protein quality control during rapid cellular proliferation and tissue differentiation [33]. Collectively, *ACTB*, *CTNNB1*, and *UBB* may act in a coordinated manner to enhance the remodeling capacity of mesenchymal cells in cattle-yak lung, providing molecular evidence for hybrid-related improvements in the structural development of cattle-yak lung.

Mesenchymal cells interact with epithelial, immune, and other cell types to maintain tissue homeostasis [34,35,36,37]. Mesenchymal cells contribute to the regulation of intracellular signal transduction in lung epithelial cells (Figure 4A). Tnc produced by mesenchymal cells was predicted to act on lung epithelial cells, which showed concurrent expression of Itgav + Itgb6 and Itgav + Itgb3 (Figure 4E). These predictions are supported by the stringent filtering of highly expressed ligand–receptor interactions and are therefore biologically meaningful, although they are derived from an in silico reference. Tenascin-C (TNC) is an extracellular matrix (ECM) glycoprotein characterized by high extensibility and mechanical resilience [38]. It contributes to tissue elasticity and mechanical stability [39] and plays an important role in epithelial–mesenchymal interactions during morphogenesis. For instance, transplantation of lung mesenchyme into non-branching epithelium induces branching formation in the trachea, implying that mesenchyme-derived factors are key drivers of branching morphogenesis in the lung. TNC remodels the ECM and facilitates airway branching morphogenesis in the embryonic chicken lung [40]. The mesenchymal expression of TNC results in ECM protein accumulation at branch tips, thereby supporting epithelial branching. These coordinated biochemical and mechanical signaling pathways enable reciprocal regulation between the epithelium and mesenchyme, ultimately shaping the final lung architecture.

Understanding cellular heterogeneity is essential for elucidating specific biological processes. Therefore, mesenchymal cells were classified into seven subtypes based on established marker genes (Figure 5B,C) and distinct transcriptional profiles derived from snRNA-seq signatures (Figure 5A). These seven subtypes were individually associated with cell migration regulation, multicellular organism development, anatomical structure morphogenesis, immune system processes, translation and peptide metabolic processes, tissue development, and blood vessel development (Figure 5D). The expression pattern and distribution of TNC were examined across all mesenchymal cell subtypes to identify the mesenchymal subtypes involved in epithelial interactions. TNC expression was notably upregulated in subtypes 0, 2, and 5. Based on marker gene profiles, these subtypes were annotated as alveolar fibroblasts, alveolar lipofibroblasts, and ductal myofibroblasts, respectively (Figure S4) [41], and were primarily associated with cell migration regulation, anatomical structure morphogenesis, and tissue development.

Finally, a key advantage of snRNA-seq in analyzing developing tissues is its ability to infer individual cell populations’ developmental trajectories through pseudotime analysis. In this study, mesenchymal cells were more abundant in cattle-yak lung than in yak lung and interacted closely with epithelial cells, suggesting a role in interstitial remodeling and morphogenesis. Although the functional importance of mesenchymal cells has been recognized, their developmental dynamics in the lungs remain poorly characterized. A pseudotime-based expression heatmap was generated to identify genes associated with mesenchymal cell state transitions, revealing 1234 genes with coordinated changes in expression across pseudotime (Figure 6B). Late-expressing genes are potential regulators of mesenchymal cell differentiation. Within these, gene cluster 2 showed a continuous increase in expression and was enriched in GO terms related to localization regulation and stimulus response and KEGG pathways associated with vascular smooth muscle contraction. Further analysis of the top enriched GO terms for cluster 2 identified *ACTB* as a core regulatory gene (Figure 6D), suggesting that this gene plays essential roles in mesenchymal cell differentiation. Collectively, these findings demonstrate the use of snRNA-seq to generate mechanistic hypotheses regarding cellular development in animal tissues.

This study has several limitations. First, the high cost of this technology and the complexity of data analysis also restrict the breadth and depth of the research to a certain extent. Cell-level statistical comparisons cannot capture inter-individual biological variation due to the limitation of biological replication, so all reported *p*-values for DEG analysis and cell-type proportion comparisons are calculated at the individual-nucleus level. Second, limited availability of validated immunofluorescence reagents, including bovine cell-specific monoclonal and polyclonal antibodies, restricted experimental validation of the identified cell types. Our current results represent exploratory molecular signatures derived from single-cell transcriptome data, and targeted experimental validation is a key direction for future follow-up investigations. Finally, only male animals were included in the present study. Therefore, our findings may not be generalizable to female yaks and cattle-yaks, and potential sex-dependent differences remain unexplored. Nevertheless, these limitations are expected to be progressively mitigated with continued advances in sequencing and validation technologies. Future work incorporating cattle controls will improve the hybrid advantage study of cattle-yak lungs.

## 4. Materials and Methods

### 4.1. Experimental Animals and Sample Collection

Lung tissue samples were obtained from three adult male yaks aged 4–6 years and three adult male cattle-yaks aged 4–6 years in Gannan at an altitude of 3000 m (Gansu province, China). All animals were provided with the same nutrition and were raised under similar environmental conditions with natural light and free access to food and water. Animals were clinically healthy at sampling time. The animals were humanely slaughtered, and small sections from the same parts of lung tissue from each animal were excised. Samples were washed twice with Dulbecco’s phosphate-buffered saline (DPBS), pooled, minced into ~1 mm^3^ pieces, and immediately frozen in liquid nitrogen. All experimental procedures were approved by the Animal Administration and Ethics Committee of the Lanzhou Institute of Husbandry and Pharmaceutical Sciences of CAAS (SYXK-2024-0044).

### 4.2. Cell Nuclear Extraction

Frozen tissues were transferred into a homogenization buffer containing 10 mM Tris, 250 mM sucrose, 25 mM KCI, 5 mM MgCl_2_, 0.1% Triton-X100 (Sigma-Aldrich, Taufkirchen, Germany), 0.5% RNasin Plus RNase (Promega Corporation, Wisconsin, USA), 1× protease inhibitor, and 0.1 mM DL-dithiothreitol. Tissues were homogenized by grinding 10–15 times to release nuclei. The homogenate was filtered through a 40-μm cell strainer (Miltenyi Biotech, Bergisch Gladbach, Germany) and centrifuged at 1000× *g* for 5 min. The pellet was resuspended in 500 μL resuspension buffer. The nuclei were counted using trypan blue (0.4%). To achieve a final iodixanol concentration of 25%, 50% iodixanol solution (500 μL) was added and thoroughly mixed. Subsequently, 1 mL of 29% iodixanol solution was carefully layered beneath the 25% mixture, followed by 1 mL of 35% iodixanol solution without mixing. The samples were centrifuged at 3000× *g* for 20 min. The nuclear layer at the interface between the 25% and 29% iodixanol layers was transferred to a new centrifuge tube. The nuclei were washed with 5 mL of resuspension buffer, gently mixed, and filtered again through a 40-μm cell strainer. After centrifugation at 500× *g* for 5 min, the nuclei were resuspended in 100 μL buffer and mixed well. Trypan blue (0.4%) was used for counting, and the ideal nuclear concentration was adjusted to approximately 1000 nuclei/μL. The nuclear suspension was kept on ice until further processing.

### 4.3. snRNA-Sequencing

Thousands of nuclei were loaded onto the 10× Genomics GemCode platform to generate single-nucleus Gel Bead-In-Emulsions (GEMs). Libraries were prepared from barcoded complementary deoxyribonucleic acid (cDNAs) using Chromium Next GEM Single Cell 3′ Reagent Kitsv3.1. Full-length barcoded cDNAs were amplified by polymerase chain reaction (PCR) to obtain sufficient material for library construction. During library preparation, P5 and P7 adapters, a sample index, and the R2 (read 2 primer sequence) were incorporated through end repair, A-tailing, adapter ligation, and PCR amplification. The final libraries contained P5 and P7 primers compatible with Illumina bridge amplification. The snRNA-seq data were deposited in the Gene Expression Omnibus under accession number GSE330031 (https://www.ncbi.nlm.nih.gov/geo/query/acc.cgi?acc=GSE330031) (accessed on 5 May 2026).

### 4.4. snRNA-Seq Data Processing

The 10× Genomics Cell Ranger software (version 6.1.0) was used to convert raw base call BCL files into FASTQ format, perform sequence alignment, and quantify gene expression. Cell Ranger uses the alignment software STAR (Spliced Transcripts Alignment to a Reference) to align Read2 to the reference genome (BosGru 3.0 Ensembl release 110). Low-quality barcodes and unique molecular identifiers (UMIs) were filtered prior to mapping to the reference genome. Reads uniquely mapped to the full transcriptome (including intronic regions) and with at least 50% overlap with gene loci were retained for UMI counting. Before quantification, UMI sequences were corrected for sequencing errors, and the EmptyDrops method was used to identify valid barcodes [42]. Cell-by-gene expression matrices were generated using UMI counting and barcode assignment.

### 4.5. Cell Clustering

Quality control, dimensionality reduction, and clustering of snRNA-seq data were performed using the Seurat package (version 5.1.0) in R [43]. Low-quality cells were excluded based on the following criteria: cells expressing <300 or >2800 genes, mitochondrial gene content exceeding 20% of total expression, or total UMI counts >7800, which may indicate low complexity, doublets, or apoptotic cells. To minimize batch effects and behavioral conditions on clustering, we used Harmony. This algorithm projects cells into a shared embedding in which cells group by cell type rather than dataset-specific conditions, to aggregate all samples. Gene expression data were normalized using Seurat’s default global normalization method (“LogNormalize”) [44]. The normalized matrix was subjected to principal component analysis (PCA), and principal components (PC) with statistical significance (*p* < 1 × 10^−5^) were selected for downstream clustering. Cell clusters were visualized using Uniform Manifold Approximation and Projection (UMAP) [45] and t-distributed Stochastic Neighbor Embedding (t-SNE), which facilitate clear separation of cell subgroups. The significance calculation method (chi-square test) was employed in the cell frequency analysis.

### 4.6. Marker Gene Identification and Differential Expression Analysis

The gene expression values in each cluster were compared with all other cells using the Seurat toolkit. Differentially expressed genes (DEGs) were defined using the following thresholds: *p*-value ≤ 0.01, log2FC ≥ 0.36, and gene detection in >25% of cells within a given cluster. Upregulated DEGs with high expression percentage were designated as cluster identification marker genes. Expression patterns of marker genes across cell populations were visualized using UMAP plots, dot plots, and heat maps. Functional enrichment analyses for Gene Ontology (GO) terms and Kyoto Encyclopedia of Genes and Genomes (KEGG) pathways were performed using g:Profiler [46,47], and the results were visualized using the ggplot2 package.

### 4.7. Ligand–Receptor Interaction Analysis

Cell–cell communication was analyzed using the CellChat (v2.1.2) [48] with default parameters. For non-model organisms, we performed homology comparisons with the mouse genes in the database, and then used the homologous genes for the analysis of cell communication in this species. Intercellular communication networks were inferred based on the expression of known ligand–receptor pairs across the identified cell types. An interaction was considered valid only when both the ligand and receptor were expressed in at least 10% of the respective tested cell populations. We screened them with a *p*-value ≤ 0.05 to evaluate whether a pair of matching receptors is significant.

### 4.8. Mesenchymal Cell Heterogeneity Analysis

Gene expression matrices corresponding to mesenchymal cells were extracted from the full dataset of all cell types. The top 1000 highly variable genes were selected for downstream analysis. The expression values were centered to a mean of zero and scaled to unit variance based on these variable genes. The expression values were expressed as follows: the normalized matrix was subjected to principal component analysis (PCA), and principal components (PC) with statistical significance (*p* < 1 × 10^−5^) were selected for downstream clustering; cell clusters were visualized using UMAP at a resolution of 0.5; DEGs specific to mesenchymal subclusters were identified, and their expression patterns were visualized using heat maps and dot plots; GO and KEGG enrichment analyses were performed on cluster-specific DEGs. These analyses followed the same workflow applied to the full expression matrix.

### 4.9. Pseudotime Analysis

Cell trajectory inference, also referred to as pseudotime analysis, was used to reconstruct differentiation trajectories and infer dynamic changes in cell subtypes during development. The cells were ordered along pseudotime based on the expression patterns of key genes, enabling the simulation of progressive transcriptional changes. Mesenchymal cell trajectory analysis was performed to characterize differentiation-related transcriptomic dynamics using the Monocle 2 package (v2.8.0). Heat maps of representative mesenchymal cell genes were generated to illustrate dynamic expression patterns along pseudotime, reflecting temporal and progressive changes in gene regulation.

## 5. Conclusions

In summary, our research unveils the paramount significance of cattle-yak lung cells and the differences between yak and cattle-yak, meticulously mapping their single-nucleus landscapes through hundreds of pivotal genes, thus offering novel avenues and methodologies for future studies on cattle-yak lung. To elucidate the functions of these key genes and to rank the importance of cells, further intensive experimental investigations are imperative to explore their biological roles.

## Figures and Tables

**Figure 1:**
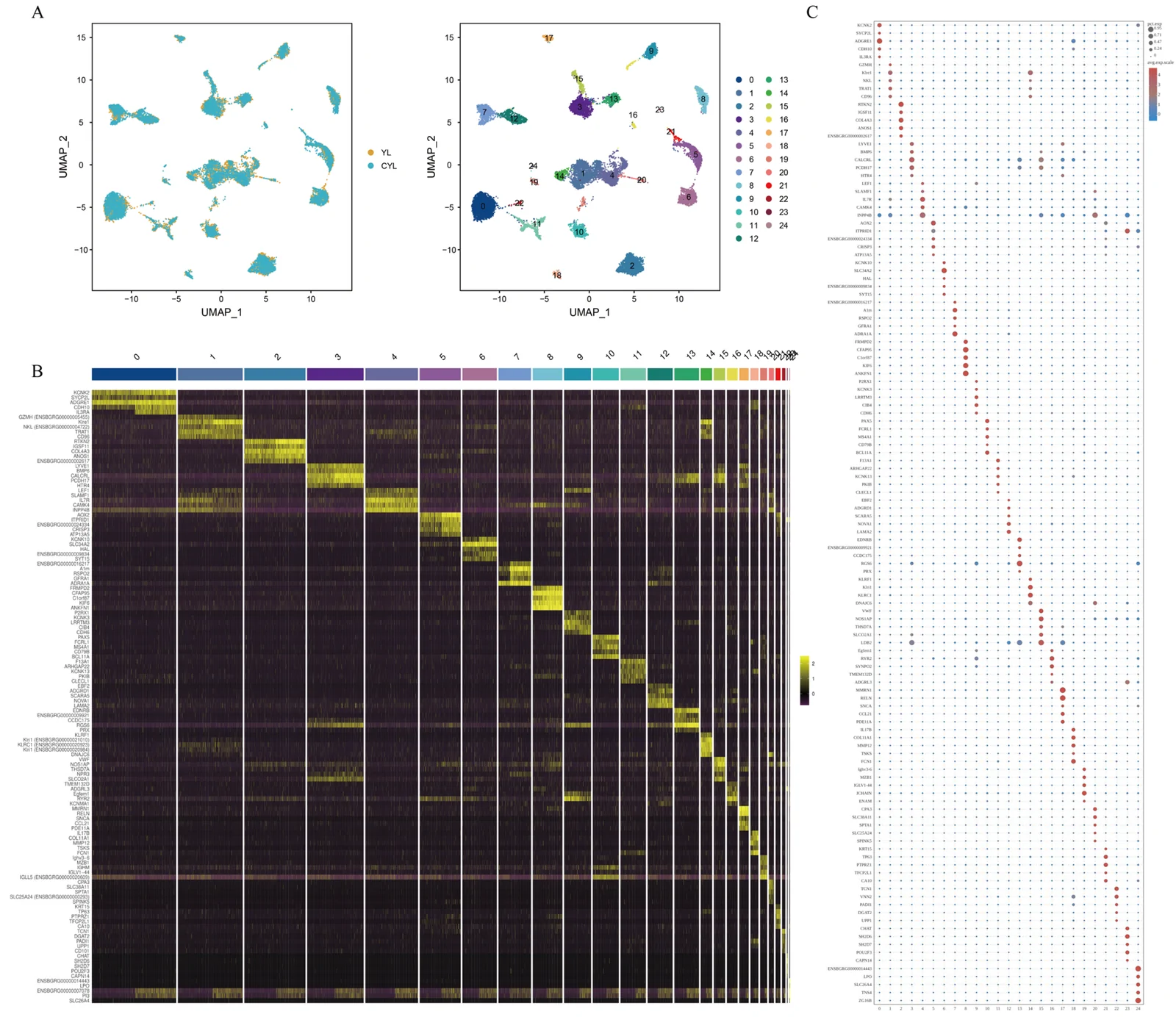
Single-nucleus RNA sequencing (snRNA-seq) identifies distinct cell clusters and signature gene profiles in lung samples from yak and cattle-yak. (**A**) UMAP scatterplot showing cell clusters in yak and cattle-yak lungs. Each point represents a single cell and is colored by cluster identity; (**B**) Dot plot showing the signature gene expression pattern across clusters. Gene expression intensity ranges from blue (low) to red (high). The percentage of cells expressing a specific gene is indicated by the size of the dot; (**C**) Heat map exhibiting distinct expression patterns of the top five most variable genes across clusters. Gene expression levels are represented by a color gradient ranging from purple (low) to yellow (high).

**Figure 2:**
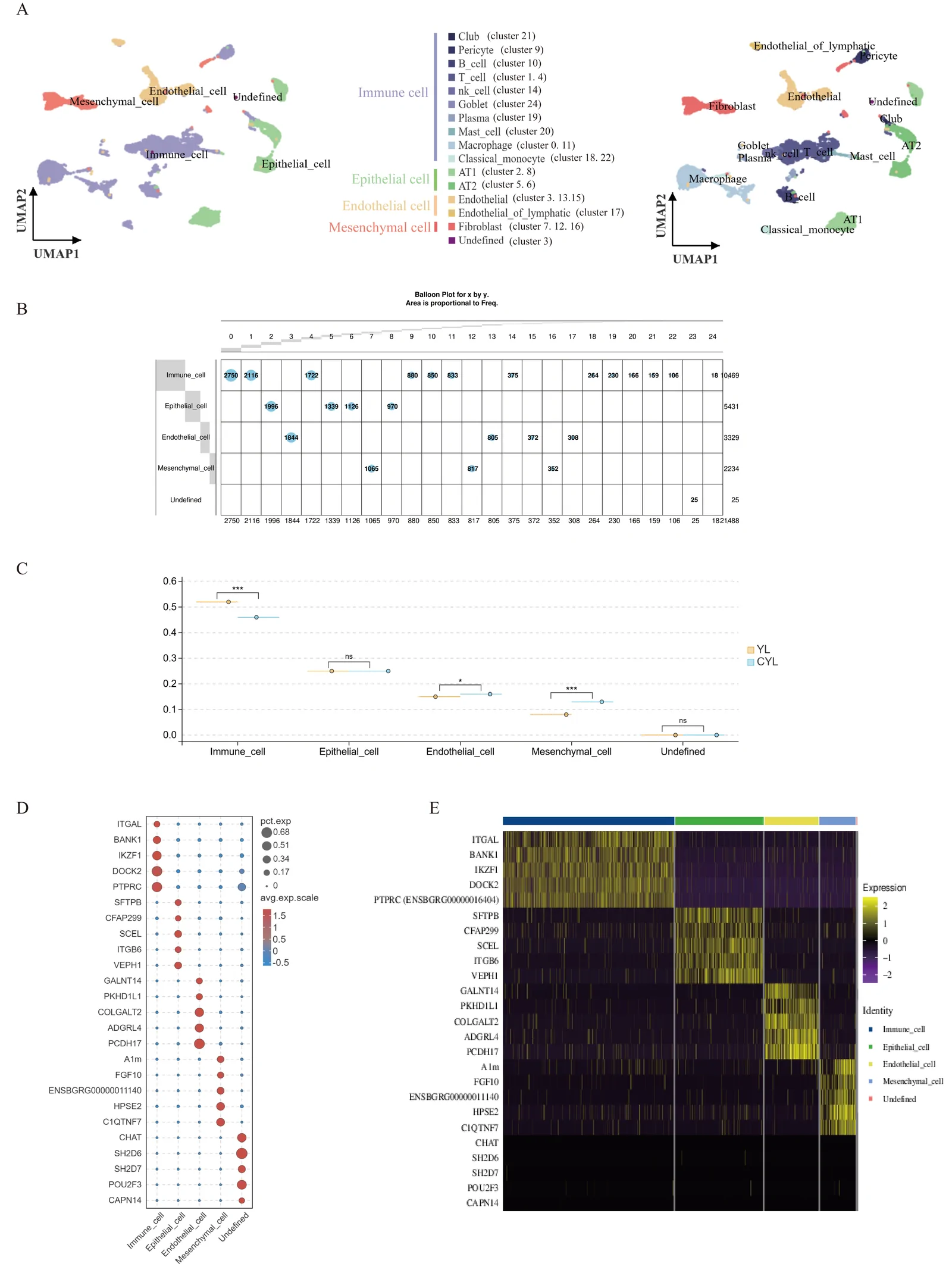
Cell types and signature gene profiles in yak and cattle-yak lung samples. (**A**) UMAP scatterplot showing the major cell types. Each point represents a single cell, colored according to cell type membership; (**B**) Balloon plot showing the distribution of cell clusters and cell types. Each column represents a cluster, with cell numbers indicated below; each row represents a cell type, with corresponding totals shown on the right side; (**C**) Comparison of cell frequency between yak and cattle-yak indicates the degree of quantitative differences in the number of cell types. The vertical axis represents the relative proportion of cell types in the corresponding groups. * *p* < 0.05, ** *p* < 0.01, *** *p* < 0.001. ns, not significant; (**D**) Dot plot showing the expression features of the selected signature genes for each cell type; (**E**) Heat map exhibiting distinct expression patterns of the top 5 most variable genes per cell type.

**Figure 3:**
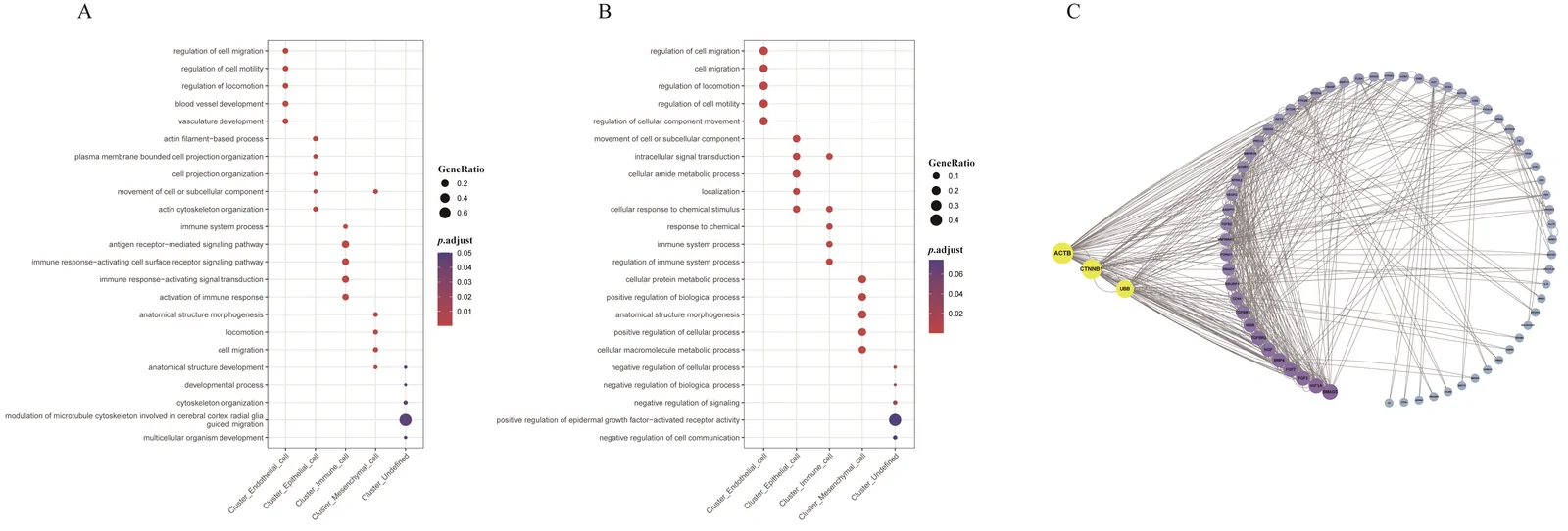
Functional enrichment analysis of lung cell types in yaks and cattle-yaks. (**A**) Bubble plot showing the top five biological process terms enriched in each cell type from the GO analysis. The bubble size indicates the gene ratio in the corresponding biological process. Adjusted *p*-values from low to high are indicated by a red-to-blue gradient; (**B**) Bubble plot showing the top five enriched biological process (BP) terms for DEGs between yak and cattle-yak from GO analysis in each cell type; (**C**) Core genes identified from the top five enriched biological process (BP) terms for DEGs between yak and cattle-yak were analyzed using STRING (12.0) and visualized using Cytoscape (3.10.4).

**Figure 4:**
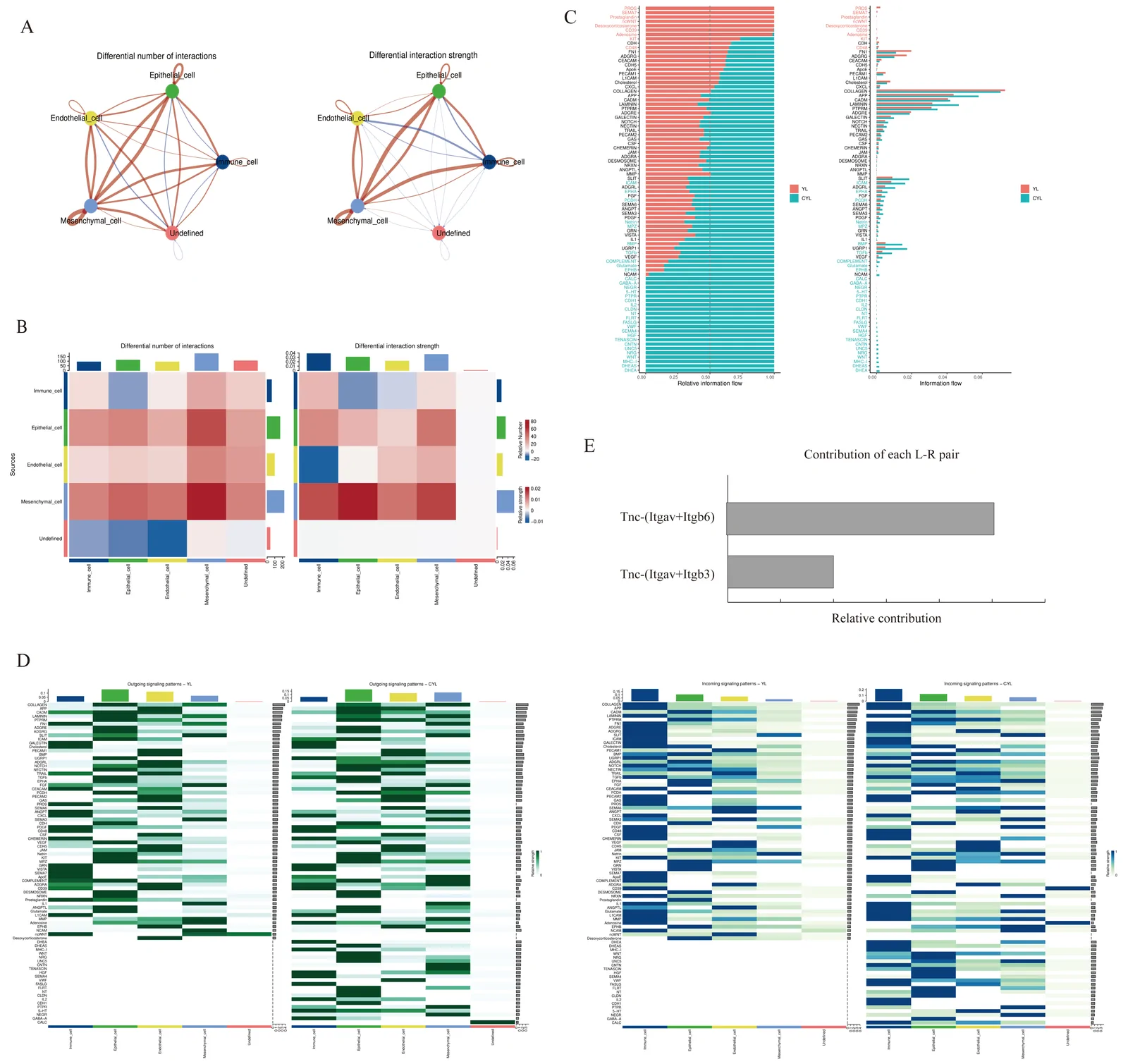
Cell-to-cell communication between mesenchymal and epithelial cells in the lung. (**A**) Network diagram illustrating differences in the number of ligand–receptor pairs between cells. Colors of the peripheral circles represent cell identities, and size reflects the number of ligand–receptor pairs associated with each cell type. The larger circles indicate a higher ratio of ligand–receptor pairs between cells. The blue and red lines represent stronger communication in yaks and cattle-yaks, respectively. The line thickness corresponds to the magnitude of change in communication strength; (**B**) Heatmap showing the differences in communication probability. The vertical and horizontal axes denote ligand and receptor cells, respectively. Color intensity reflects differences in communication probability, with deeper shades indicating stronger communication. The top and right bar plots display the cumulative differences in communication probabilities along the vertical and horizontal axes, respectively; (**C**) Bar chart depicting significant cell–cell contact signaling pathways between mesenchymal and epithelial cells inferred using CellChat. Relative signaling strength in yak is shown in red, and that in cattle-yak is shown in green. Labels are colored red when significantly enriched in yak relative to cattle-yak, and green if significantly enriched in cattle-yak. Pathways are ordered according to the differences in the relative information flow between the yak and cattle-yak; (**D**) Heatmap showing the relative signaling intensities of significantly enriched pathways identified by CellChat. The top bar plot indicates the total signaling strength of all displayed pathways within each cell type, whereas the right gray bar plot summarizes the total signaling strength of all displayed cell types in each pathway; (**E**) Relative contribution of individual ligand-receptor pairs to the overall TENASCIN signaling communication network in the lungs.

**Figure 5:**
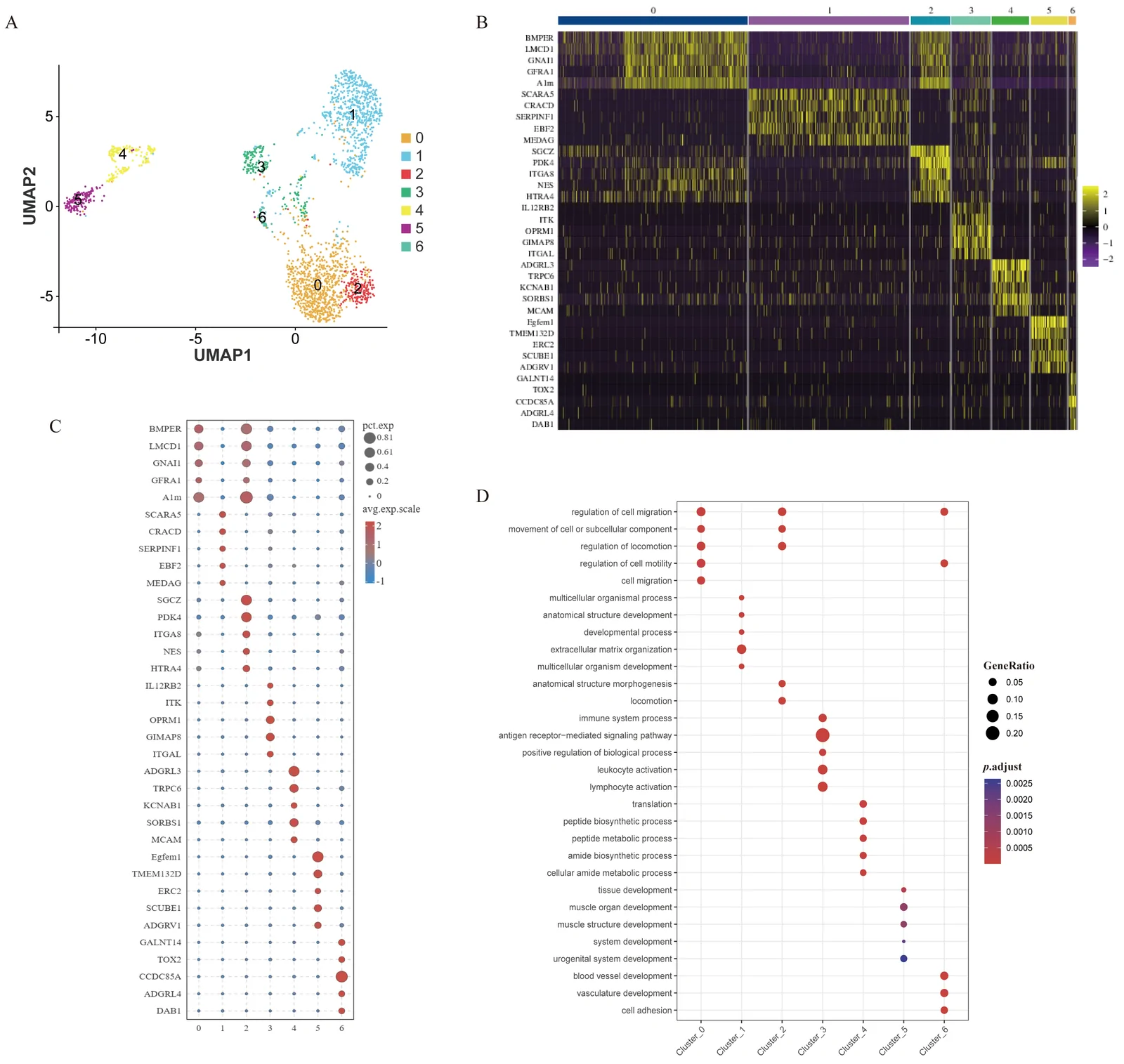
Heterogeneity of mesenchymal cells in yak and cattle-yak lungs. (**A**) UMAP scatterplot showing mesenchymal cell subtypes. Each point represents a single color-coded cell based on its cell subtype membership; (**B**) Heatmap exhibiting distinct expression patterns of the top 5 most variable genes across mesenchymal cell subtypes; (**C**) Dot plot showing the expression features of the selected signature genes for each mesenchymal cell subtype; (**D**) Bubble plot showing the top five enriched biological process (BP) terms of each mesenchymal cell subtype from GO analysis. The bubble size indicates the ratio of genes in the corresponding biological process. Adjusted *p*-values from low to high are indicated by a red-to-blue gradient.

**Figure 6:**
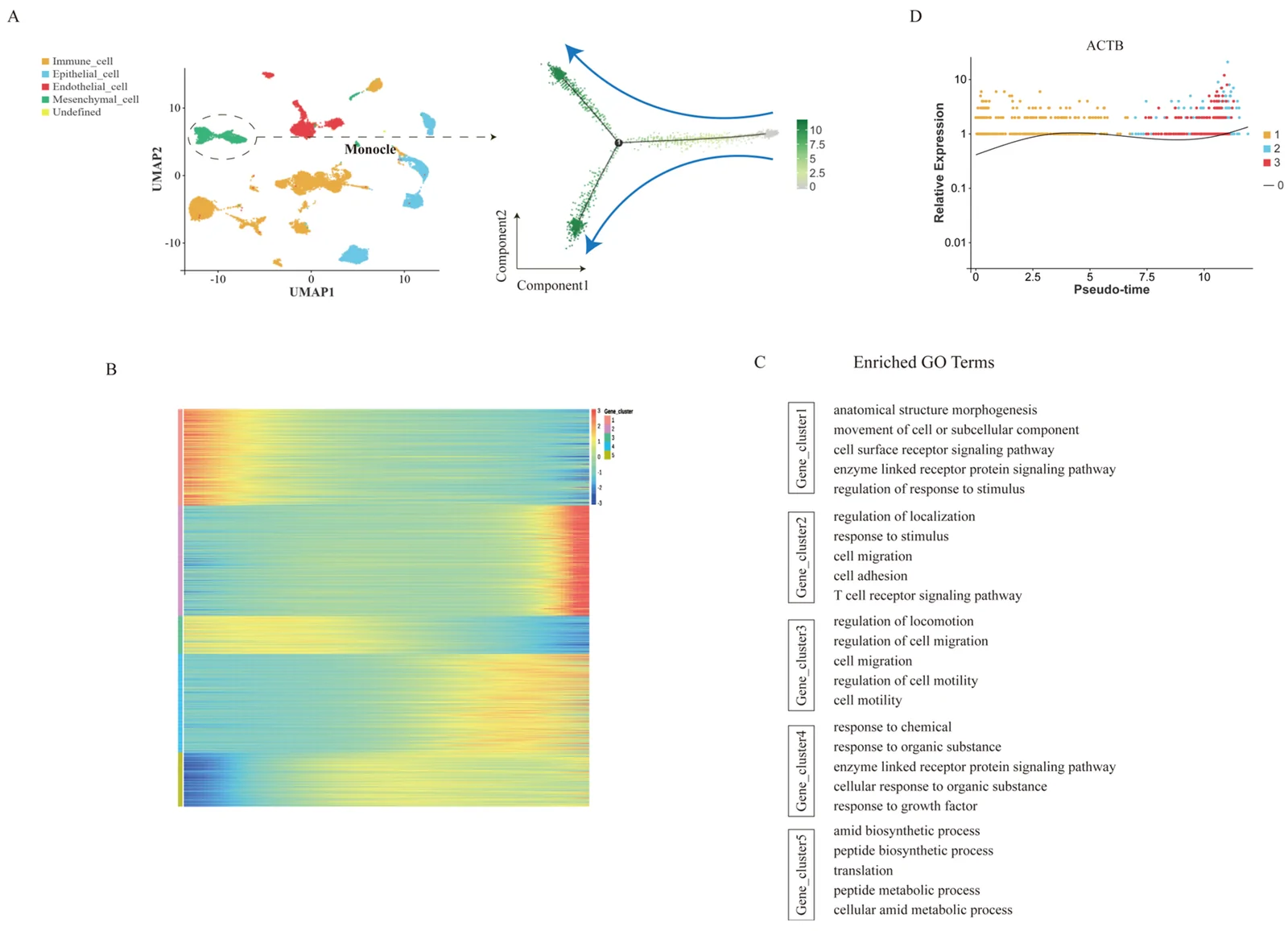
Pseudotime analysis of mesenchymal cells in the lung. (**A**) Pseudotime trajectory of 2234 mesenchymal cells generated using Monocle software. Each point represents a unique cell, and the cyan arrows indicate the inferred developmental pseudotime directions of the mesenchymal cells; (**B**) Heatmap showing scaled and centered gene expression (Z-scores) of 1234 dynamically expressed genes over the developmental trajectory of mesenchymal cells as determined by snRNA-seq; (**C**) Top 5 GO terms enriched in each group by fold enrichment; (**D**) Scatterplots arranged along pseudotime exhibiting the expression dynamics of core mesenchymal cell genes.

## Data Availability

The snRNA-seq data were deposited in the Gene Expression Omnibus under accession number GSE330031 (https://www.ncbi.nlm.nih.gov/geo/query/acc.cgi?acc=GSE330031) (accessed on 5 May 2026).

## Supplementary Materials

The following supporting information can be downloaded at: https://www.mdpi.com/article/10.3390/ijms27198531/s1.
